# Enabling fully automated insulin delivery through meal detection and size estimation using Artificial Intelligence

**DOI:** 10.1038/s41746-023-00783-1

**Published:** 2023-03-13

**Authors:** Clara Mosquera-Lopez, Leah M. Wilson, Joseph El Youssef, Wade Hilts, Joseph Leitschuh, Deborah Branigan, Virginia Gabo, Jae H. Eom, Jessica R. Castle, Peter G. Jacobs

**Affiliations:** 1 Artificial Intelligence for Medical Systems (AIMS) Lab, Department of Biomedical Engineering, Oregon Health & Science University, Portland, OR USA; 2 Harold Schnitzer Diabetes Health Center, Oregon Health & Science University, Portland, OR USA

**Keywords:** Type 1 diabetes, Machine learning

## Abstract

We present a robust insulin delivery system that includes automated meal detection and carbohydrate content estimation using machine learning for meal insulin dosing called robust artificial pancreas (RAP). We conducted a randomized, single-center crossover trial to compare postprandial glucose control in the four hours following unannounced meals using a hybrid model predictive control (MPC) algorithm and the RAP system. The RAP system includes a neural network model to automatically detect meals and deliver a recommended meal insulin dose. The meal detection algorithm has a sensitivity of 83.3%, false discovery rate of 16.6%, and mean detection time of 25.9 minutes. While there is no significant difference in incremental area under the curve of glucose, RAP significantly reduces time above range (glucose >180 mg/dL) by 10.8% (*P* = 0.04) and trends toward increasing time in range (70–180 mg/dL) by 9.1% compared with MPC. Time below range (glucose <70 mg/dL) is not significantly different between RAP and MPC.

## Introduction

Closed-loop systems for automated insulin delivery are now the standard of care in type 1 diabetes (T1D) management, helping people living with diabetes better manage their glucose while reducing burden^1,2^. However, currently available systems are hybrid in nature and still require the person to count carbohydrates and manually announce meals to the system. Postprandial glucose control is substantially improved when meal insulin is delivered before meal intake.

Carbohydrate counting is challenging and people living with T1D are oftentimes inaccurate in estimating carbohydrate intake. Current carbohydrate counting methods require a level of numeracy and literacy that might be a barrier for some people with diabetes^3^. Gillingham et al. showed that 49% percent of meals with <30 g of carbohydrates are overestimated while the majority (64%) of large carbohydrate meals (≥60 g) are underestimated^4^. Inaccurate carbohydrate estimations that are used for calculation of prandial insulin are associated with high prevalence of postprandial hyper- and hypoglycemia, even with hybrid insulin delivery systems^5,6^.

Several approaches to automated meal detection have been described in the literature, which generally use continuous glucose measurements (CGM) and insulin delivery data, and in some cases physical activity data. Some of the approaches of previously published work include fuzzy logic^7^, Kalman filtering^8–10^, super-twisting observer combined with Kalman filtering^11^, probabilistic models^12^, quantification of the difference between predicted glucose using an autoregressive or other models vs. measured CGM values^13^, and glucose increase detection^14^. Smartwatch gesture-based meal reminders have also been proposed for improved postprandial glycemic control^15^. Sensitivity varies greatly depending on the datasets used and the study protocols, reaching values greater than 90% in some cases, though the sensitivity and specificity of many of previously published algorithms have been validated only in silico. For example, the automated meal detection algorithm presented by Corbett et al.^16^ used a clustering method to estimate the probability of a meal occurring based on prior meal patterns. The algorithm recognizes a meal pattern and doses a priming dose, demonstrating an improvement in time in range from 52 to 57%; however, these results are only provided for an in silico trial. Several prior manuscripts have reported on the effectiveness and safety of using machine-learning-based automated meal detection within automated insulin delivery systems in clinical trials. Recently, Tsoukas et al. reported on a fully automated system that utilized a Kalman filter model-based automated meal detection and multiple hormone delivery, including pramlintide and insulin in response to meal detections. They showed that a fully automated pramlintide plus insulin delivery system was not inferior to an insulin-only hybrid automated insulin delivery system^17^. In this work, we contribute a new machine learning model for meal detection and meal size estimation that is incorporated into a robust insulin delivery system and tested in humans to assess feasibility and safety of semi-automated meal insulin delivery with minimal user intervention. The algorithm has a sensitivity of 83.3%, false discovery rate of 16.6%, and mean meal detection time of 25.9 min. When comparing the benefit of dosing bolus insulin in response to a detected meal with the robust insulin delivery system vs. adjusting insulin infusion rate using a hybrid insulin delivery system with no meal announcement, there is no significant difference in incremental area under the curve of glucose. However, the robust insulin delivery system with automated meal detection significantly reduces time above range (glucose >180 mg/dL) by 10.8% (*P* = 0.04) without significantly increasing risk of postprandial low glucose (glucose <70 mg/dL).

## Results

### Participants information

**Fig. 1:**
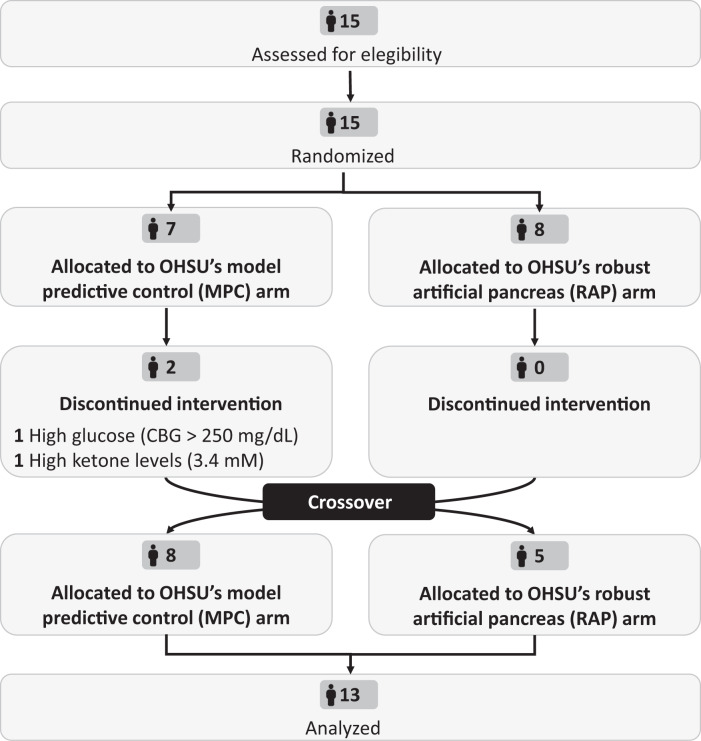
CONSORT flow diagram. Flow diagram of the progress through the phases of the randomized, single-center crossover trial to compare OHSU’s MPC vs. RAP insulin delivery systems.

Fifteen adults were enrolled to participate in this study between December 2021 and March 2022. Table 1 summarizes participants’ characteristics and Fig. 1 shows the study CONSORT diagram. The carbohydrate content of the study meals chosen by participants ranged from 45 to 66 g. Two participants withdrew from the study due to (1) high glucose (CBG > 250 mg/dL) and (2) high ketone levels (3.4 mM) following a meal during the model predictive control (MPC) algorithm arm. The markedly elevated ketone level was attributed to the participant’s ketogenic diet and the participant was withdrawn from the study. These participants were not included in the analysis. There were no serious adverse events during the study.

**Table 1:** Baseline characteristics of eligible participants (*N* = 15).

Demographics	
Age, years	37.6 ± 10.4
Biological sex, *N* (%)	Female: 9 (60.0)
	Male: 6 (40.0)
Weight, Kg	85.0 ± 18.8
Ethnicity (self-identified), *N* (%)	White: 13 (86.6)
	Black: 1 (6.7)
	American Indian: 1 (6.7)
Clinical data	
HbA1c, %	6.8 ± 0.5
Duration of diabetes, years	24.8 ± 9.0
CGM use, *N* (%)	Dexcom: 10 (66.6)
	Medtronic: 3 (20.0)
	Libre: 1 (6.7)
	None: 1 (6.7)

### Study outcomes

Supplementary Table 2 shows meal insulin recommended by the robust artificial pancreas (RAP) system for true positive meal detections and the adjustments made by participants or clinical team after discussing the safety of the proposed adjustment and reaching an agreement about the final insulin amount to be delivered. The study participant or the investigator made small adjustments to the insulin bolus 54.5% of the time when a true positive detection occurred. However, the recommended meal insulin amount was reduced only once, by an amount of 1.5 units. The average absolute change in bolus insulin across all participants was very low at 0.35 units.

Mean postprandial incremental area under the curve (iAUC) of glucose was lowest with RAP but not significantly different from the MPC automated insulin delivery algorithm (−23.6, 95% CI: −120.6 to 73.4 mg h/dL; *P* = 0.63). Time above range (TAR) 70–180 mg/dL was significantly reduced with RAP by 10.8% compared with MPC (time above range 95% CI: 0.02 to 24.4%; *P* = 0.04). Time in range (TIR) 70–180 mg/dL was higher with RAP by 9.1% compared with MPC, but the observed difference was not statistically significant (TIR 95% CI: −1.5 to 22.9%; *P* = 0.09). Time below range (TBR) at less than 70 mg/dL and time below 54 mg/dL were slightly higher with RAP, but not statistically different from MPC (TBR < 70 mg/dL 95% CI: −0.7 to 2.3%; *P* = 0.52 and TBR < 54 mg/dL 95% CI: −0.4 to 1.3%; *P* = 0.46).

**Fig. 2:**
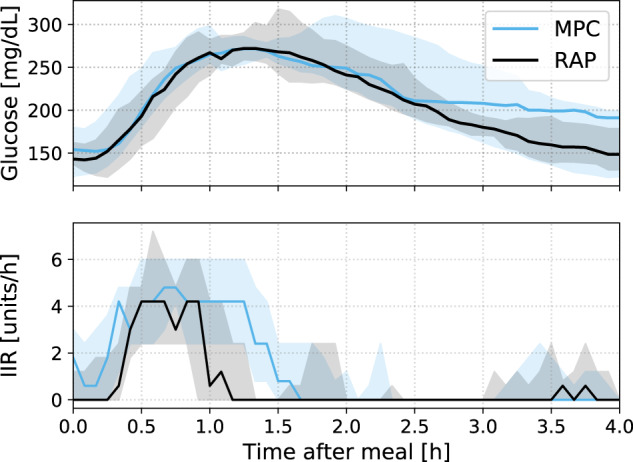
Comparative postprandial sensor glucose (top) and insulin infusion rate (IIR)(bottom) during the MPC and RAP study arms following breakfast meal from the 13 participants who completed the study. Median and interquartile range are shown.

Postprandial CGM and insulin (median and interquartile range) during the four hours after meal are shown in Fig. 2. Glucose traces for MPC and RAP were similar for the first two hours after the meal. After two hours, glucose traces were lower during the RAP arm compared with the MPC arm such that at four hours after the meal, the median glucose was substantially lower for RAP compared with MPC (148.5 vs. 191.0 mg/dL, Fig. 2). RAP dosed more insulin (8.7 ± 3.2 vs. 7.6 ± 3.3 units) and delivery occurred sooner after the meal compared with MPC, which tended to deliver over a longer period after the meal (Fig. 2, lower panel).

The machine learning model automatically detected 83.3% of the 24 meals consumed during the RAP studies (95% CI 62.6 to 95.2%). The false detection rate was 16.6% (95% CI 4.7 to 37.4%). The reported false detection rate includes a meal detection corresponding to when a rescue carbohydrate was consumed by the participant. In future versions of the RAP algorithm, the RAP algorithm will ignore rapid glucose rises caused by rescue carbohydrates that were reported to the system. Overall meal detection time was 25.9 ± 0.9 minutes. The accuracy of the meal detection and classification algorithm was close to the predictions made in the pre-study simulations. The average in silico sensitivity for meals with carbohydrate content between 40-80 g, which were the categories of self-selected study meals during the clinical trial, ranged from 79.0 to 90.0% with a probability detection threshold *P*_TH_ = 0.86 used in the study (see Supplementary Fig. 2). Similarly, in silico meal detection time was calculated to be 27.5 ± 4.8 min. The in silico false discovery rate was 10.0% when we did not include false detection caused by low glucose rescue carbohydrate intake as false detections. Thus, the meal detection accuracy results obtained in this study matched almost exactly pre-clinical in silico validation results demonstrating that in silico metabolic simulators based on ordinary differential equations can be effectively used to estimate performance prior to a real-world clinical study in humans.

## Discussion

The use of RAP which includes an automated meal detection and meal size estimation machine learning algorithm resulted in a statistically significant reduction in postprandial time above range following unannounced meals in adults with T1D, and a modest (though not statistically significant) improvement in TIR. These results are relevant given that time below range was not significantly increased by RAP when compared with an MPC algorithm. Two carbohydrate treatments were administered during the RAP arm of the study in response to the system’s predicted low glucose alert while no rescue carbohydrate treatments were given during the MPC arm. There was one event in which a participant experienced glucose below 70 mg/dL following a meal bolus during the RAP arm. For this event, glucose at meal detection time was 200 mg/dL and rising, and the low blood glucose index (LBGI)^18^ values ranged from 0.0 within the 0–2 h period following the meal bolus to 4.3 over the 0–4 h after the bolus.

The performance of the meal detection algorithm was very close to the performance observed in silico during pre-clinical algorithm validation. The algorithm had high sensitivity and low false discovery rate. There were four false detections, but one of them occurred due to a sharp glucose rise in the morning at about 8:30 AM, which is a pattern with a high likelihood of being associated with a breakfast meal. Another false detection was associated with a sharp rise in glucose following the consumption of a rescue carbohydrate consumed in response to a hypoglycemia treatment. Given the possibility that a false meal detection could increase the risk of hypoglycemia, we calculated LBGI following false meal detections to see whether delivering insulin in response to a false alarm led to increased risk of low glucose(LBGI ≥ 5.0)^11^. The calculated LBGI was zero in three out of the four false detection cases indicating that no low glucose events had occurred. For the fourth false detection case, the LBGI ranged from 0.94 to 3.3. For this case, the false detection occurred in response to a rescue carbohydrate that was consumed by the participant, which was misinterpreted as a meal by the RAP algorithm. In future usage, the RAP automated meal detection will be disabled following the report of rescue carbohydrate consumption.

The results of this study indicate that if a meal is accurately detected within 25 to 30 min of the meal occurring and dosed a percentage of the nominal required prandial insulin, time in hyperglycemia can be significantly reduced and there is no significant increased risk of postprandial hypoglycemia. Delayed detection is a consequence of delays in carbohydrate absorption and inherent delay in glucose measured in the interstitium relative to blood glucose. We found that, on average, a detectable glucose rise due to meal intake only occurs after 20 minutes of consuming a meal, and this delay is also dependent on the meal composition. For instance, one of the meals that the algorithm was unable to detect had a high fat content which caused a slower rise in glucose and a delayed glucose peak. It’s unlikely that insulin could be dosed for a meal using a CGM-based automated detection any sooner than 20 min after the meal was consumed because of the delay in rise in glucose. Results presented here show that dosing meal insulin approximately 25 min after the meal using a missed meal detection and classification algorithm provides benefit compared with depending on the control algorithm to naturally respond to a meal.

One limitation of this study was the small sample size. However, even with this small sample size, a benefit was shown in terms of reducing high glucose levels for the RAP compared with MPC. Another limitation of this study is that the meal detection required participant confirmation. The participant or investigator were allowed to modify the amount of insulin recommended by the RAP algorithm; thus, these adjustments might have impacted the glucose control performance metrics. The modifications made by the participant or investigator were small (0.35 units on average) indicating that the participant may have been ‘fine tuning’ the insulin bolus rather than correcting a grossly inaccurate recommendation. The insulin was reduced by the participant or investigator only once indicating that in 90.9% of the cases, the RAP was being more conservative and delivering slightly less than what the participant wanted to deliver. This indicates that the system is performing in a safe way to avoid over-delivery of insulin in response to the RAP meal detection algorithm. One reason why participants may have chosen to dose more insulin than recommended by the RAP algorithm could have been a result of the fact that the RAP algorithm was designed to dose only a fraction of meal insulin (up to 75%), since it was being dosed approximately 25 min after the meal event occurred. Participants may have wanted to dose the full 100% of the meal insulin dosage, even though it was being dosed late. A larger study without meal confirmation by participants is needed to assess whether a fully automated insulin delivery system provides significant benefits in terms of reduction in postprandial incremental area under the glucose curve and increase in time in target range, which were defined as primary outcomes of this feasibility study.

This study provides evidence that a machine-learning-based model for meal detection and carbohydrate content estimation can be integrated into automated insulin delivery systems to improve postprandial glucose control. Moreover, we demonstrated that a meal detection model trained on in silico data obtained from validated T1D simulators achieves nearly identical performance in this clinical study thereby providing further evidence in the support of use of metabolic simulators for developing and evaluating tools in advance of clinical studies.

## Methods

### Meal detection and carbohydrate estimation model development

We obtained datasets from simulations performed using two validated T1D simulators: (1) The UVA-Padova simulator, which is approved by the Food and Drug Administration (FDA) for in silico pre-clinical validation; and (2) a published open-source simulator developed by Oregon Health & Science University (OHSU)^19^. We simulated 199 virtual subjects for 14 days using real-world meal scenarios collected from previous studies (low-carbohydrate diet: 46.6 ± 27.1 g, high-carbohydrate diet: 72.5 ± 29.3 g)^13,20^. Glucose control was simulated using OHSU’s MPC automated insulin delivery algorithm^21^. Thirty three percent of the meals were given without a corresponding insulin bolus.

A total of 32 features were derived from the two-hour history of CGM and insulin measurements obtained prior to a meal prediction. Examples of features used included average glucose, glucose rate of change, insulin availability^22–24^ one hour before prediction time, and prediction time (i.e., hour of day). Since the prediction hour (0–23) is a cyclical feature, we transformed it into two dimensions using cosine and sine operations as follows: $\cos \left( {2\pi \frac{{\rm{hour}}}{{24}}} \right),\;\sin \left( {2\pi \frac{{\rm{hour}}}{{24}}} \right)$. A descriptive list of input features used for meal detection and carbohydrate content estimation is provided in Supplementary Table 1. We used these features as inputs to a multioutput neural network with fully connected layers (Supplementary Fig. 1 shows high-level architecture of the model). The designed network has three shared hidden layers for processing the input features with 512, 32, and 16 nodes, and two dedicated branches for meal detection (i.e., binary classification) and carbohydrate estimation (i.e., multiclass classification) with a 16-node hidden layer per branch. For multiclass classification, meal sizes were categorized into five groups as follows: [0,20) g, [20,40) g, [40,60), [60,80), and 80+ g. Hyperparameters of the model including number of layers and nodes per layer were determined through cross-validation. L1 regularization with penalty constant of 1e−6 was used in all hidden layers. All weights were randomly initialized using Xavier uniform initializer^25^, and bias were initially set to zero. Adam optimizer with constant learning rate of 1e−4 and recommended values for the rest of parameters^26^ was used to minimize binary and categorical cross-entropy losses for detection and classification outputs, respectively. Training was done with mini batches of size 128. Detection loss and classification loss were equally weighted. For carbohydrate content estimation, the samples in the training dataset were weighted to account for imbalance in the dataset and for penalizing overestimation. Early stopping was implemented to help prevent overfitting. Meal detection was determined to have occurred if it exceeded a probability threshold of *P*_TH_ = 0.86 as determined through simulations. The size of the meal was determined by the meal class node that yielded the highest probability from the neural network. This study evaluates the first version of the meal detection algorithm.

### Automated insulin delivery systems

The OHSU’s MPC algorithm^21^ uses a glucoregulatory model to predict glucose outcomes over a predicted horizon, and mathematically solves for the optimal insulin dose schedule across the control horizon to bring a person to a target glucose level. The MPC algorithm includes a Kalman filter, which uses the difference between sensor-measured glucose levels and model predictions to update the physiologic model states at each timestep for personalized predictions. The MPC strategy has been previously described in people living with T1D^21,27–33^.

The RAP system uses a modified MPC algorithm that includes the machine leaning model described herein for missed meal insulin detection. The missed meal insulin detection alert notified the participant through a smartphone app if the probability of a meal detection exceeded a threshold of 0.86 as determined through simulations (see Supplementary Fig. 2). The screens in the app used to notify the participant of a missed meal insulin detection are shown as Supplementary Fig. 3. These screens provide a recommended amount of insulin to dose in response to the detected meal. The recommended amount of meal insulin to deliver is based on (1) the output of the missed meal insulin detection algorithm, (2) the size of the meal estimation as determined by the missed meal insulin detection algorithm, (3) the person’s carbohydrate ratio, and (4) the time when the person responded to the alert. Since the meal insulin is being dosed after the meal was consumed, only a fraction of the person’s typical meal insulin is recommended to be dosed in response to the missed meal insulin detection algorithm. The percentage of the meal insulin recommended to be dosed is a function of the expected time after which the meal was consumed such that the meal insulin dosed is reduced by 1% for every 1 min after which the meal was presumed to occur. We determined through simulations (see “Results”) that the missed meal detection algorithm triggered on average about 25 min after a meal was consumed. Therefore, if the missed meal insulin detection algorithm detected the meal and notified the study participant and they acknowledged the alert immediately, then the meal insulin dosed would be 75% of the meal insulin determined by the person’s carbohydrate ratio and the expected meal amount as determined by the missed meal insulin detection algorithm. However, if the participant waited 20 min to respond to the alert, then the amount of recommended insulin would be further reduced by 20%. For instance, if the missed meal detection probability exceeds the threshold of 0.86, and the meal size predicted by the algorithm is 30 g, and the person’s carbohydrate ratio is 1:10 g, and the participant responded to the alert immediately, then the person would be recommended to receive 0.75 × 30 g/10 g/unit = 2.25 units of insulin. Additional features of the RAP system include automated physical activity detection and classification as measured from a smart fitness watch (Polar M600); however, these features were not used in this study.

Both MPC and RAP systems have safety features including predicting low glucose suspend insulin delivery using a long- short-term memory (LSTM) neural network^24^, maximum insulin dosing based on users’ total daily insulin requirement, switch to background basal insulin if CGM or pump communications are disrupted, and smartwatch on/off wrist detection algorithm. Both control systems run on a smartphone app called iPancreas that has been used to evaluate other automated multi-hormone delivery systems^20,34^.

### Study design and participants

This paper reports on the outcomes of a single-center, crossover trial designed to compare the glucose control following unannounced meals achieved using OHSU MPC vs. RAP automated insulin delivery systems. Individuals with diagnosis of T1D for at least one year, aged 18–65 years, current use of an insulin pump for at least three months with stable insulin pump settings for longer than 2 weeks, HbA1c ≤ 10.5% at screening, and total daily insulin requirement less than 139 units/day were eligible for inclusion. Exclusion criteria included pregnancy or intention to become pregnant, current use of a glucose lowering medication other than insulin, and use of oral or parenteral corticosteroids.

Pre-clinical validation of the MPC and RAP automated insulin delivery systems was done using computer simulations. The accuracy of the machine learning model in detecting meals was also retrospectively validated on a real-world large dataset from 150 closed-loop participants (age 29 ± 16 years; 66 females, 47 males, 37 records with unknown biological sex; 15 ± 12 years since T1D diagnosis) from the Tidepool Big Data Donation Program (Tidepool.org, Palo Alto, CA) that contains more than 115,000 meals.

This study was conducted under U.S. Food and Drug Administration–approved investigational device exemption, approved by the OHSU Institutional Review Board, and registered on ClinicalTrials.gov (NCT05083559, first posted on October 19, 2021).

### Procedures

Written informed consent was obtained from all participants during the study screening visit. Participants underwent two treatment visits at OHSU for evaluating glucose control following unannounced meals with OHSU MPC and RAP insulin delivery systems in a randomized order. For each intervention visit, participants arrived at approximately 7:00 AM and were monitored through the afternoon and discharged before dinner. Participants wore an Omnipod pump (Omnipod Insulet Corporation, Acton, MA, USA) to deliver insulin and a Dexcom G6 CGM to measure glucose (DexCom, Inc., San Diego, CA, USA). The RAP system captured activity data (i.e., heart rate and accelerometry) through a Polar M600 watch (Polar Electro Inc., Bethpage, NY, USA) worn by the participants. After a run-in period of two hours whereby the participants used the automated insulin delivery systems, participants ate self-selected meals at 10:00 AM for breakfast. The carbohydrate content of the meals allowed per protocol was 45–120 g. During the RAP study visit only, participants were given a second meal four hours later at 2:00 PM when the study staff considered it appropriate to further evaluate the accuracy of the meal detection algorithm. Self-selected breakfasts were identical across both study sessions. A meal bolus was not given before any meals. CBG and blood ketone measurements were taken every 30 min until discharge for safety purposes only and not to inform control decisions of the automated insulin delivery systems under evaluation. During the RAP study visit, the meal detection algorithm was used to identify a missed meal bolus. If the RAP system detected a missed meal, the system sent an alert to the participant indicating that a meal was detected, and the system also provided an estimate of the carbohydrate content of the detected meal. Participants were required to acknowledge the alert. Participants could modify the carbohydrate content that was estimated by the RAP system. The modified carbohydrate estimation was used to calculate the meal bolus, which was dosed by the RAP system via an Omnipod insulin pump. A study investigator evaluated the meal insulin dose prior to delivery, considering insulin availability (I), time since last meal, and glucose trend, and modified the dose if appropriate for participants safety.

All data collected during the study including glucose sensor data, insulin data, physical activity data, and meal data, were aggregated in real time by the OHSU iPancreas app and stored for remote monitoring and further analysis on a cloud-based database hosted on an OHSU-managed secure server called iPancreas Guidance Remote Monitoring (GRM) hosted by Amazon Web Services.

### Outcomes

The primary endpoints prespecified for this study were (1) the incremental area under the curve iAUC of glucose (calculated using the trapezoidal rule) and (2) percent of time with glucose sensor in target range between 70 and 180 mg/dL in the four hours following unannounced breakfast meals. Post-prandial iAUC is defined as the area of the post-prandial glucose response curve above the baseline glucose (i.e., sensor glucose at the start of the meal). iAUC is useful to control variations in baseline glucose across participants. iAUC is different from the typical area under the curve (AUC) calculation in which the area is calculated relative to zero rather than relative to the baseline glucose. As secondary clinical outcome metrics, we assessed postprandial glucose control metrics including percent time with sensor glucose below range (<70 mg/dL) and percent time with sensor glucose above range (>180 mg/dL).

We also analyzed the performance of the meal detection machine learning model in detecting unannounced meals using sensitivity, false discovery rate, and meal detection time. These metrics were calculated based on participant confirmation of meal detection alerts and data on mealtime records entered by a study investigator to the GRM. The primary and secondary endpoints reported here were calculated only using glucose and insulin data collected over the four hours following the breakfast meal. However, the accuracy of the meal detection algorithm was evaluated using both breakfast and lunch meals.

To contextualize the performance of the machine learning model, we compared the accuracy results from this study with pre-clinical in silico results obtained using the FDA approved UVA/Padova and OHSU T1D simulators.

### Statistical analysis

We estimated the differences between the MPC and RAP systems in postprandial glucose control using a fixed effect in a standard crossover model with a random participant effect. We used mixed effects linear regression for the analysis of postprandial iAUC; and mixed effects beta regression for TIR, TAR, and TBR. A *P* value <0.05 was considered statistically significant.

We used the Clopper–Pearson method to calculate the exact 95% confidence interval of the sensitivity and false discovery rate of the machine learning model in detecting meals. Other reported metrics, including the meal detection time, are expressed as mean ± standard deviation (SD) unless otherwise noted.

Data processing and statistical analyses were carried out using Python 3.7 and R 4.1.3

## Supplementary information

Supplementary Material

## Data Availability

Data might be made available for researchers with ethical approval from the OHSU Institutional Review Board. Interested parties should contact P.G.J. and C.M.L. (jacobsp@ohsu.edu, mosquera@ohsu.edu).
