# Supplementary material for: Enabling fully automated insulin delivery through meal detection and size estimation using Artificial Intelligence
Source: NPJ Digit Med. 2023 Mar 13;6:39. doi: 10.1038/s41746-023-00783-1 (PMC10011368; doi:10.1038/s41746-023-00783-1)
Supplement: Supplementary file 1 — Supplementary Material [file 41746_2023_783_MOESM1_ESM.pdf]

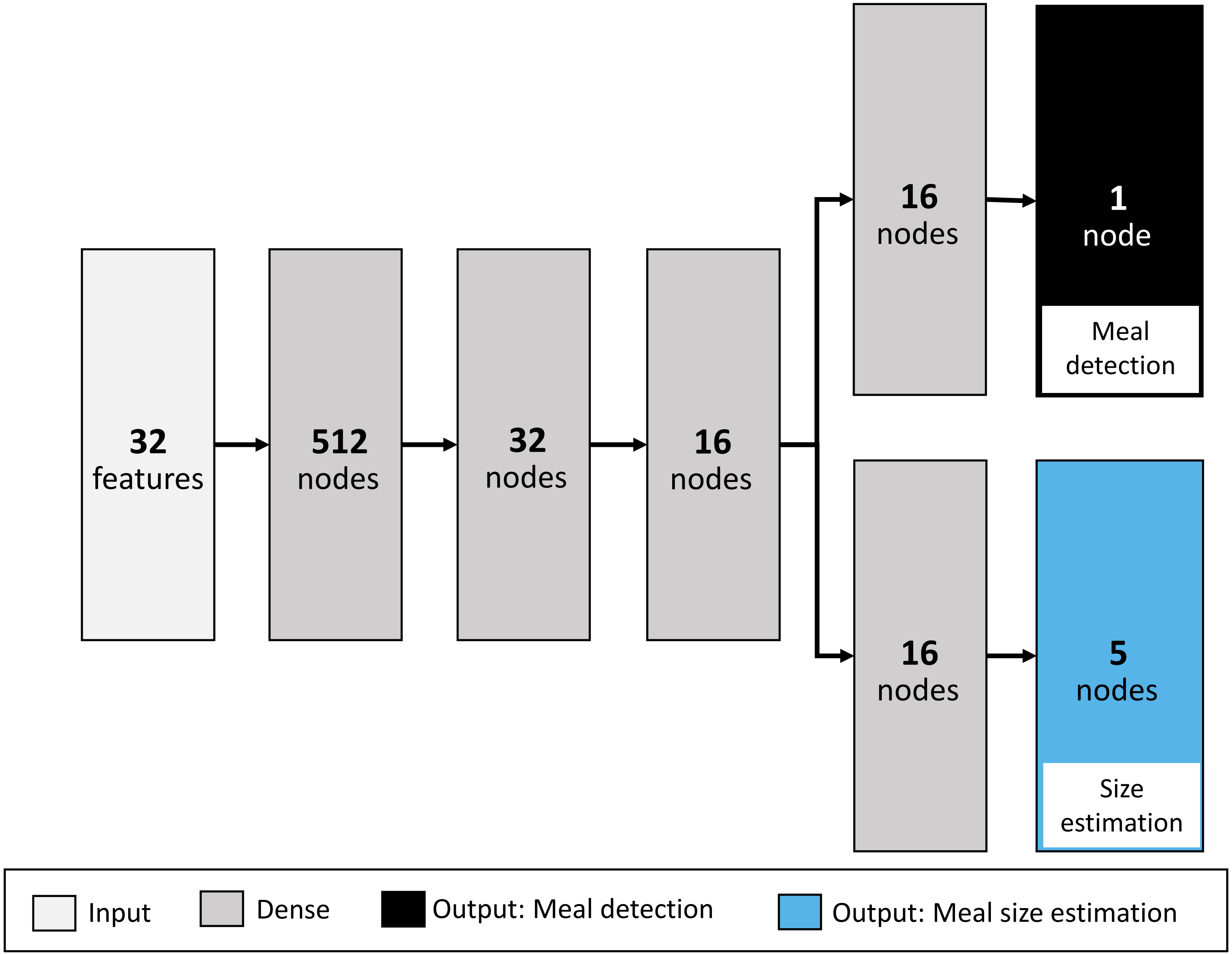

Supplementary Fig. 1 Architecture of the multioutput neural network designed for meal detection and meal size estimation.

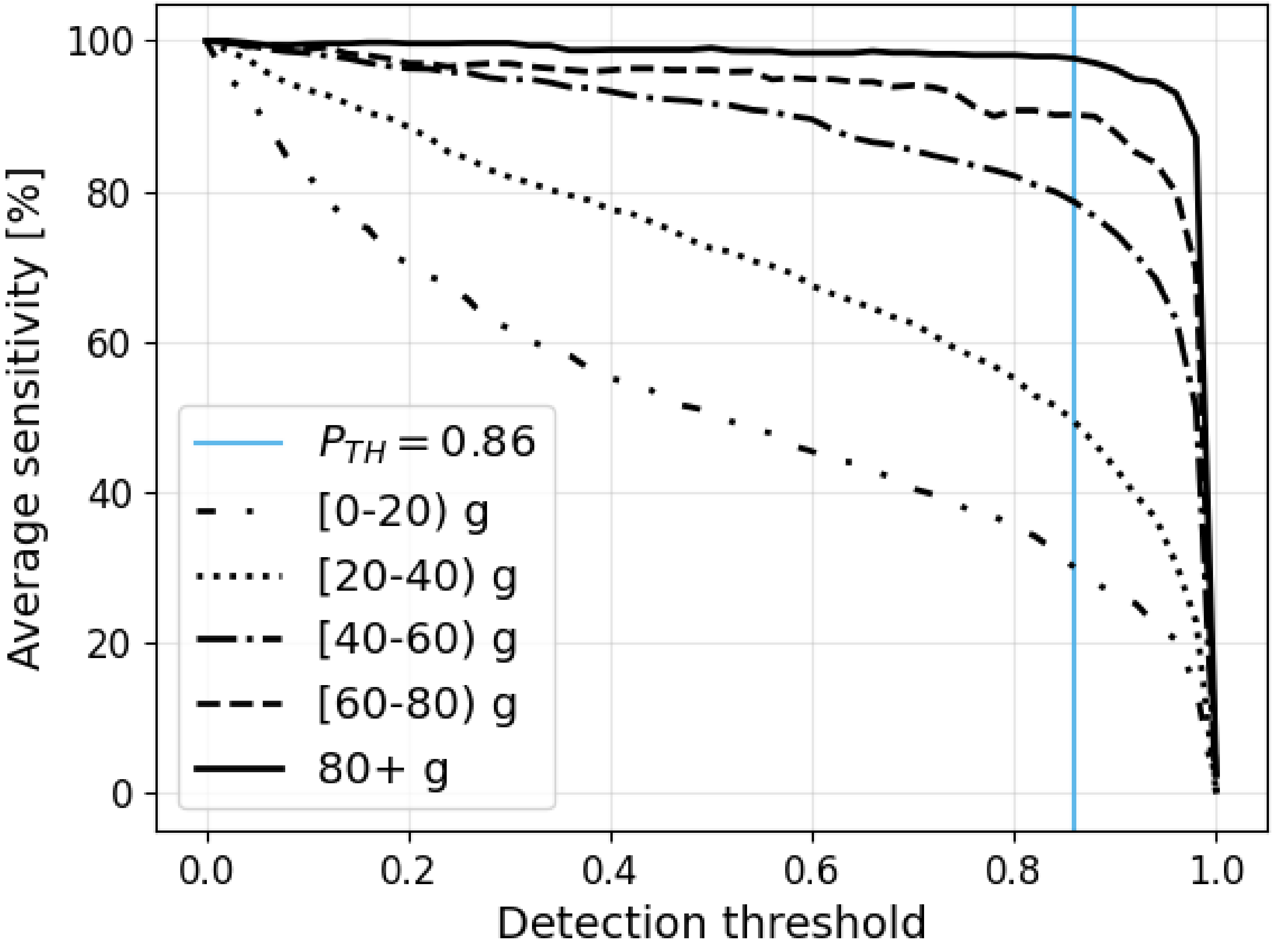

Supplementary Fig. 2 *In silico* sensitivity of the proposed machine learning meal detection algorithm for meals with varied carbohydrate content.

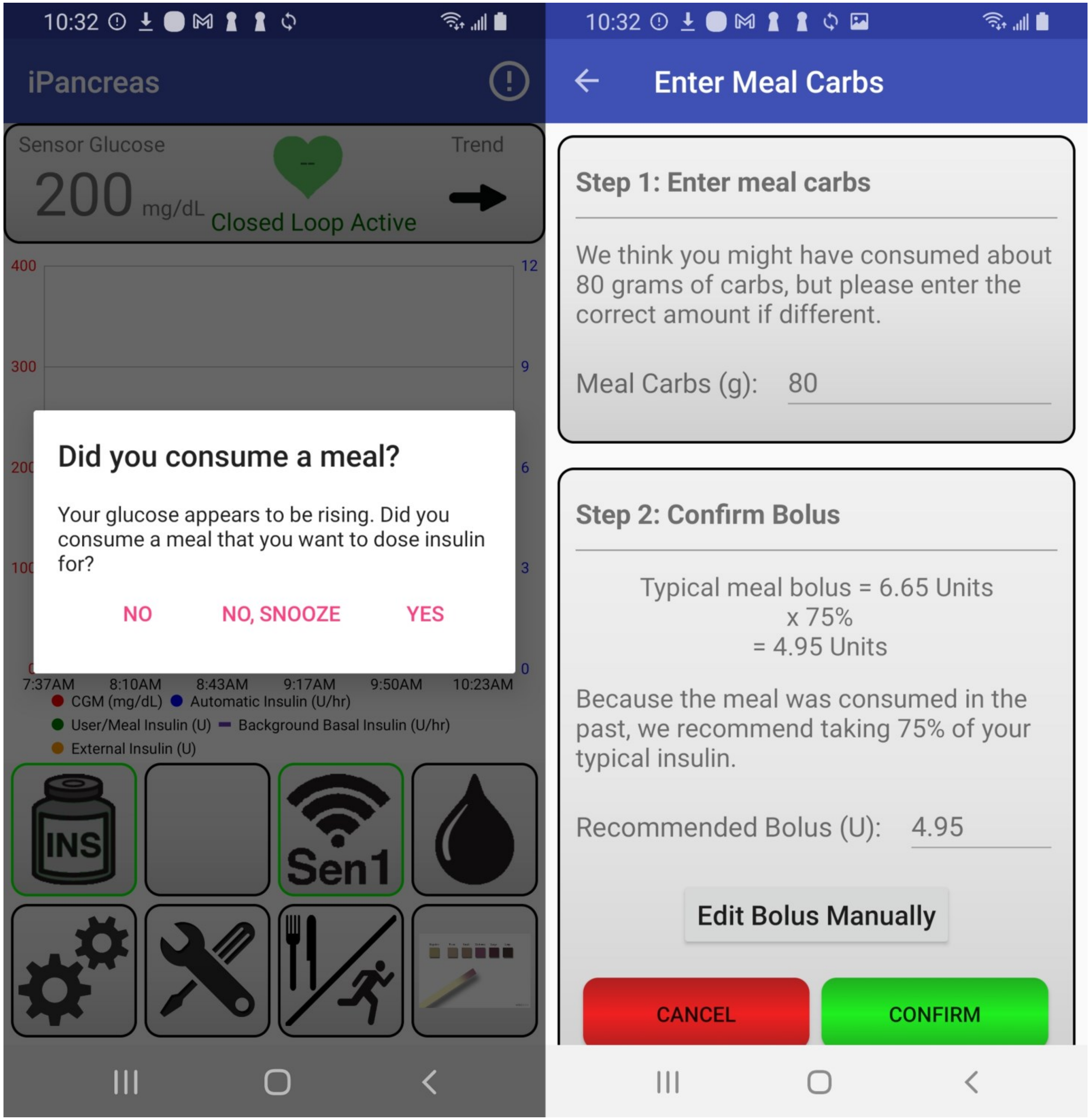

Supplementary Fig. 3 *iPancreas* screens for notifying the participant of a missed meal insulin detection.

Supplementary Table 1. Features used for meal detection and meal size estimation.

Description	Number of features	Specification / Calculation
**Glucose features**		
Glucose at the time of prediction	1	$f_1 = CGM_k$
Time series of glucose measurements corresponding to one-hour worth of data prior to the prediction time	12	$f_{2-13} = CGM_{k-h}$ $h \in \{1,2,3,\dots,12\}$
Glucose rate of change (GROC)[1] at the time of prediction	1	$f_{14} = \frac{CGM_k - CGM_{k-1}}{\Delta t}$
Average GROC during the hour prior to prediction.	1	$f_{15} = \frac{1}{13}\sum_{h=0}^{12} GROC_{k-h}$
Count of GROC values over the hour prior to prediction that are greater than pre-defined thresholds.	8	$f_{16-24} = \sum_{h=0}^{12} f(GROC_{k-h})$ $f(ROC_{k-h}) = \begin{cases} 1, & if\ ROC_k > th \\ 0, & otherwise \end{cases}$ $th \in \{0.0,0.5,1.0,1.5,2.0,2.5,3.0,6.0\}$
Average glucose over one hour prior to prediction time	1	$f_{24} = \frac{1}{13}\sum_{h=0}^{12} CGM_{k-h}$
Average glucose calculated from two hours to one hour prior to prediction time	1	$f_{25} = \frac{1}{12}\sum_{h=13}^{24} CGM_{k-h}$
Difference in average glucose during the last half hour vs. the preceding half hour	1	$f_{26} = \frac{1}{6}\sum_{h=7}^{12} CGM_{k-h} - \frac{1}{7}\sum_{h=0}^{6} CGM_{k-h}$
Average difference between glucose over 30 minutes prior to prediction with respect to the glucose value exactly 30 minutes prior to prediction time	1	$f_{27} = \frac{1}{6}\sum_{h=0}^{5} (CGM_{k-h} - CGM_{k-6})$
Binary value, set to 1 if the ROC at prediction time is greater than 5 mg/dL/min	1	$f_{28} = \begin{cases} 1, & if\ GROC_k > 5 \\ 0, & otherwise \end{cases}$
Binary value, set to 1 if the ROC at prediction time is greater than 7 mg/dL/min	1	$f_{29} = \begin{cases} 1, & if\ GROC_k > 5 \\ 0, & otherwise \end{cases}$
**Insulin features**		
Insulin availability calculated 60 minutes prior to prediction time	2	$f_{30} = I_{k-12}$
**Time features**		
Time of day (hour, 0-23)	2	$f_{31} = \cos\left(2\pi \frac{hour}{24}\right)$ $f_{32} = \sin\left(2\pi \frac{hour}{24}\right)$

[1] GROC is computed using second-order central differences in the interior points and first order forward or backwards differences at the boundaries.

Supplementary Table 2. RAP bolus recommendations and bolus delivered. True positive detections shown (11 out of 13 breakfast meals were detected).

Participant ID	RAP recommended bolus [U]	Delivered bolus [U]	Absolute difference
**Breakfast**			
501	4.30	5.00	0.70
502	6.55	6.55	0.00
504	5.80	5.80	0.00
505	7.50	6.00	-1.50
507	2.75	2.75	0.00
508	3.35	5.00	1.65
509	4.10	4.50	0.40
510	5.50	5.50	0.00
512	3.15	5.00	1.85
513	3.30	4.00	0.70
514	3.80	3.80	0.00
**MEAN ± STD**	**4.55 ± 1.56**	**4.90 ± 1.09**	**0.35 ± 0.91**
